# What matters to you? A qualitative study on the lived experiences of community-living individuals with dementia

**DOI:** 10.1093/geroni/igaf122.2236

**Published:** 2025-12-31

**Authors:** Rashmita Basu, Donna Roberson

**Affiliations:** East Carolina University, Greenville, North Carolina, United States; East Carolina University, Greenville, North Carolina, United States

## Abstract

With Alzheimer’s Disease and Related Dementia (ADRD) increasing in the U.S., more research is needed to deliver patient-centered care. However, recruiting people with ADRD is challenging. This study examines what matters to individuals living with dementia in their community through the conceptual lens of person-centered care. We conducted seven qualitative interviews with individuals who have early or mid-stage dementia and live in the community. Two researchers used an inductive line-by-line approach to perform thematic data analysis independently and then as a team. The analysis revealed one overarching theme with four sub-themes. The overarching theme was holistic person-centeredness in dementia care. The four sub-themes were: (1) understanding the interplay between disease and daily-life activities; (2) participating in meaningful activities based on one’s preferences; (3) incongruence between the person with dementia’s preferences, needs, and the care received from healthcare providers; and (4) concerns of lost income due to job loss, burdening the family, and lack of comprehensive dementia care supports. There is a clear incongruence between individual preferences and needs and a demand to operationalize person-centeredness in dementia care, including the healthcare provider’s role. This group of participants wants the “incongruence” resolved to improve person-centered care. Healthcare providers should focus on encouraging individuals with dementia to participate in meaningful activities in daily routines. As a start to person-centeredness in care delivery, healthcare providers need to know and share resources based on their patients’ needs and preferences for care.

